# Light-driven polymer recycling to monomers and small molecules

**DOI:** 10.1038/s41467-024-46656-3

**Published:** 2024-03-20

**Authors:** Laura Wimberger, Gervase Ng, Cyrille Boyer

**Affiliations:** Cluster for Advanced Macromolecular Design and School of Chemical Engineering, The University of New South Wales, 2052 Sydney, NSW Australia

**Keywords:** Photocatalysis, Polymerization mechanisms, Polymers

## Abstract

Only a small proportion of global plastic waste is recycled, of which most is mechanically recycled into lower quality materials. The alternative, chemical recycling, enables renewed production of pristine materials, but generally comes at a high energy cost, particularly for processes like pyrolysis. This review focuses on light-driven approaches for chemically recycling and upcycling plastic waste, with emphasis on reduced energy consumption and selective transformations not achievable with heat-driven methods. We focus on challenging to recycle backbone structures composed of mainly C‒C bonds, which lack functional groups i.e., esters or amides, that facilitate chemical recycling e.g., by solvolysis. We discuss the use of light, either in conjunction with heat to drive depolymerization to monomers or via photocatalysis to transform polymers into valuable small molecules. The structural prerequisites for these approaches are outlined, highlighting their advantages as well as limitations. We conclude with an outlook, addressing key challenges, opportunities, and provide guidelines for future photocatalyst (PC) development.

## Abstract (web summary)

Chemical recycling of plastic enables renewed production of pristine materials, but generally comes at a high energy cost. This review focuses on light-driven approaches for chemically recycling and upcycling plastic waste, with emphasis on reduced energy consumption and selective transformations of challenging to recycle backbone structures.

## Introduction

**Fig. 1:**
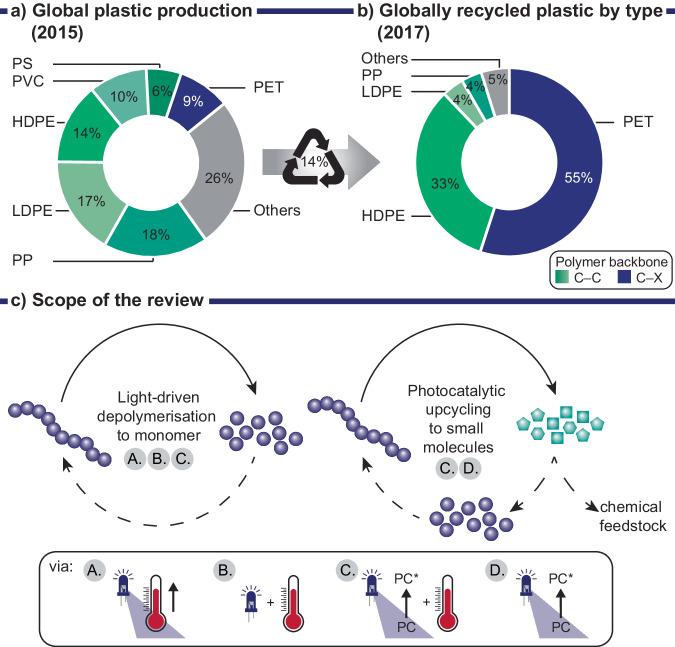
Analysis of global plastic production, recycling efforts and scope of this review. **a** Global plastic production of which 14% is recycled^1^. **b** Globally recycled plastics by type of polymer in 2017^14^: HDPE – high-density polyethylene, LDPE – low-density polyethylene, PP – polypropylene, PVC – polyvinylchloride, PS – polystyrene, PET – polyethylene terephthalate. Types of polymers are color coded based on their backbone structure where shades of green indicate carbon-carbon (C–C) bonds and blue indicates carbon-heteroatom (C–X) bonds. **c** Light driven depolymerization to monomer and photocatalytic upcycling to small molecules using various stimuli: A. Light-induced heating, B. Light and heat, C. Photocatalytic approach in combination with heating, D. Purely photocatalytic approach.

Only 14% of plastic worldwide is recycled (Fig. 1), 14% is incinerated and the remaining quantity ends up in landfill (40%) or is leaked into the environment (32%)^1^. Needless to say, mankind has accumulated a formidable amount of plastic waste since its establishment in the mid-20th century. The widespread plastic pollution, with microplastic having reached remote areas like the Antarctic, severely impacts the health of our environment, and some long-term effects are still unknown^2–8^. In order to tackle this immense pollution problem large leaps in technology and recycling approaches are required to move towards a more circular approach where waste provides the new feedstock to produce materials.

Aside from energy recovery by incineration, mechanical plastics recycling is the main current approach for resource recovery, where plastics are sorted, washed, ground and melted into pellets for a new application^9^. While desirable from an emissions and energy perspective, this approach generally results in impaired material properties (down-cycling) compared to virgin-grade plastic, preventing it from being used in medical or food packaging applications^10^. Therefore, the regeneration of monomers in the recycling process proves advantageous, as it opens up a wide range of potential applications for the resulting materials. This strategy, commonly referred to as chemical recycling, is gaining traction as a complimentary approach to conventional mechanical recycling. However, chemical recycling often faces a significant drawback in the form of high energy consumption, especially in processes like pyrolysis, which demand temperatures in the range of 300 to 500 °C^11^. Pyrolysis is able to recover mixed plastic waste by applying heat under an inert atmosphere, resulting in the production of monomers, gases, oils, and char^11^. In some cases, e.g., for LDPE, using chemically recycled monomers from pyrolysis for renewed plastic production was found to be less energy consuming and lower in CO_2_ emissions than the production of virgin plastic^12^. This makes chemical processing a valuable alternative to landfill or incineration for otherwise difficult to recycle plastic waste.

While chemical recycling may produce a variety of products including monomers, upcycling typically involves creating higher-value products. However, assessing product values can be challenging for early-stage technologies, leading to the generous use of the term for processes that yield defined products.

Even in forerunning economies such as in the EU where 35% of plastics are recycled (2020)^13^ there is a big discrepancy between the different types of plastics produced and the available recycling technologies that are specific to different types of polymers. Globally the largest component of recycled plastics is made up of polyethylene terephthalate (PET, 55% in 2017)^14^ which only makes up 9% (in 2015)^1^ of the overall plastics production. Easier to recycle—or chemically transform—polymers contain heteroatoms in their backbone (C‒X) offering a selective site which may be cleaved by processes like solvolysis. Most consumed polymers, such as polypropylene (PP) and polyethylene (HDPE and LDPE), however, contain C‒C backbone structures which are much more challenging to chemically recycle as selective activation of C‒H or C‒C bonds is required.

Aside from achieving selective chemical transformations, several other factors of the recycling process i.e. collection, sorting, and purification pose significant challenges but are not the focus of this review. For example, the chemical recycling to monomer for polymethyl methacrylate (PMMA) has been long known, but the recycling of this polymer is limited by its use in specialty applications making collection a significant hurdle^15^.

Light offers a less energy intensive approach for chemical recycling as well as unique selectivity and reactivity by using photocatalysts. This perspective gives an overview of the recent developments that use light to chemically re- or upcycle a variety of different polymers. Other approaches of polymer degradation/upcycling use thermal, electrocatalytic or biological approaches and are not discussed here. We focus predominantly on the most challenging to recycle polymers, made up of C–C backbones. However, some promising approaches to degrade C–X polymers, such as epoxy phenolic resins, are also discussed. By categorizing the different methods based on photocatalytic approaches and material requirements our aim is to draw a clear picture of the *status quo* for using light to drive carbon backbone-based polymer re- and upcycling. For more detailed insight on some of the concepts discussed here we point to reviews covering controlled depolymerization (thermal)^16^, heterogeneous catalysis^17,18^, photocatalytic upcycling^19^, and photoreforming^20^.

The review is split into two main sections: Light-induced depolymerization to monomer and photocatalytic small molecule generation i.e., upcycling (Fig. 1c). In the first section we distinguish between uncontrolled and controlled depolymerization whereby the latter discusses polymers derived from reversible-deactivation radical polymerization (RDRP). Both methods rely on the input of heat which is either generated by light or supplied in addition to light irradiation. The photocatalytic upcycling methods predominantly avoid use of heat and are discussed according to their underlying principles: proton coupled electron transfer (PCET), ligand to metal charge transfer (LMCT), hydrogen atom transfer (HAT), or side chain induced backbone scission. We briefly touch on photoreforming approaches that use C–C polymers to generate hydrogen fuel. Finally, we give some perspective on the challenges of the individual approaches, assess their potential and the value of small molecule products.

### Depolymerization

#### Thermal depolymerization

**Fig. 2:**
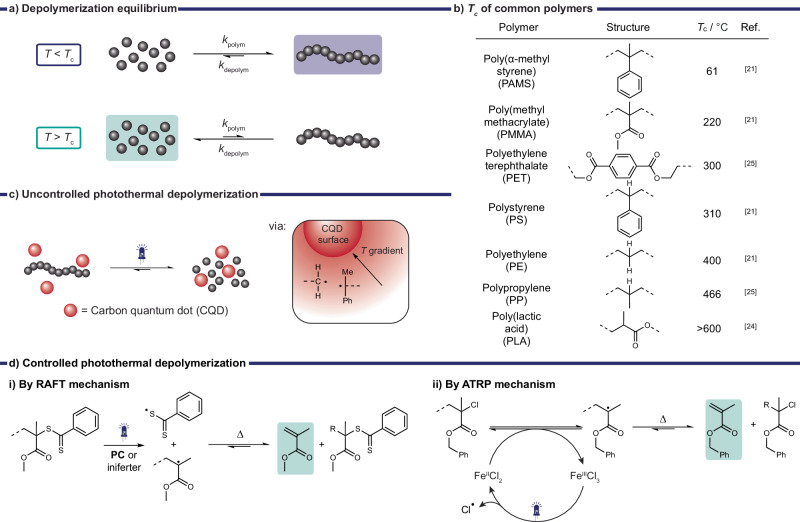
Overview of the depolymerization strategies. **a** Thermal depolymerization equilibrium. **b** Ceiling temperature (*T*_c_) of common polymers. The value of poly(lactic acid) (PLA) is extrapolated as decomposition reactions occur before depolymerization. **c** Uncontrolled photoinduced thermal depolymerization^27^. **d** Controlled photothermal depolymerization by (i) reversible addition-fragmentation chain-transfer (RAFT) photoiniferter or photocatalytic (PC) processes^30–32^, or (ii) atom transfer radical polymerization (ATRP) mechanisms^34^.

The reversibility of a polymerization process, also referred to as depolymerization, is primarily related to the ceiling temperature (*T*_c_)^21^. When the ceiling temperature is attained, the rates of both polymerization and depolymerization become equal (Fig. 2a). Exceeding *T*_c_ leads to a preference for depolymerization over polymerization, causing the regeneration of monomers due to a shift in the equilibrium between monomers and active propagating chains.

The *T*_c_ value is determined by increasing the temperature in the polymerization process and in some cases by extrapolation^16^. This makes *T*_c_ highly dependent on the applied polymerization conditions. Factors such as concentration, steric hindrance around the propagating radical, and solvent have a significant influence on *T*_c_^21–23^. Hereby, reduced concentrations lower the *T*_c_ and bulky substituents also tend to lower the *T*_c_. The stability of the (de)propagating radical also drastically affects *T*_c_ whereby more stable radicals i.e., tertiary radicals, have lower *T*_c_ values. For example, styrene has a relatively high *T*_c_ of 395 °C (secondary radical) compared to 61 °C of its methyl substituted analogue α-methylstyrene (PAMS, tertiary radical).

The *T*_c_ of most commodity plastics exceeds 300 °C (Fig. 2b) making their depolymerization either extremely energy intensive or impossible when the decomposition occurs at temperatures below *T*_c_, for example for poly(lactic acid) (PLA)^21,24,25^. Additionally, achieving selective initiation to access the depolymerization equilibrium is challenging and requires temperatures well above *T*_c_ for chemical recycling by pyrolysis. Though *T*_c_ provides insight on the temperature dependence of the (de)polymerization equilibrium, it should be treated with care due to its dependence on a multitude of factors, especially concentration. This also means that depolymerization can be achieved at temperatures lower than the reported *T*_c_ as demonstrated recently for PLA (*T*_c_ > 600 °C in bulk → *T*_c_ 119‒138 °C in 0.5 M solution)^26^.

#### Uncontrolled photoinduced thermal depolymerization

Bulk heating of polymers including mixtures remains the most common strategy for depolymerization. However, the use of ambient conditions during this process may result in oxidized by-products due to the unselective formation of reactive intermediates^27^. Stache and coworkers proposed a selective chemical recycling strategy that used light responsive carbon quantum dots (CQDs) to generate local temperature gradients (Fig. 2c). Using broad spectrum white light under ambient conditions, various polymers, such as PAMS, PMMA, PS, PET, and PLA, were effectively depolymerized to their respective monomers in good yields (43‒95%, Table 1). The localized generation of heat by CQDs significantly reduced oxidized byproducts (<1%) compared to conventional bulk heating (290 °C) which produced 16% by-products for the depolymerization of PAMS. The reduction of by-products was attributed to a decreased concentration of reactive radical intermediates generated by the light-induced temperature gradients. Exquisite spatial control of this method also allowed selective depolymerization of mixed polymers by interspersion of CQDs into the polymer with lower *T*_c_ (PAMS-PMMA, PAMS-PS).

**Table 1:** Reported examples of photoinduced thermal depolymerization^a^

Entry	Polymer	Light source	Conditions	Monomer/Product	Time	Yield	Refs.
1	PLA	Broad-spectrum white light	Solvent cast film, under vacuum, 0.5 eq. ZnO, CQD	Latic acid	4 h	95%	^27^
2	PET	Simulated sunlight (600 mW cm^‒2^)	PET (0.25 wt%) in ethylene glycol, choline phosphate (0.05 wt%), CNT-PDA (0.005 wt%)	BHET	4 h	82%	^28^
3	PAMS	Broad-spectrum white light	Solvent cast film, CQD	AMS	30 min	66%	^27^
4	PMMA	Broad-spectrum white light	Solvent cast film, under vacuum, CQD	MMA	4 h	64%	^27^
5	PS	Broad-spectrum white light	Bulk, under vacuum, 0.07 eq. BaO, CQD	Styrene	2 h	43%	^27^

*AMS* α-methyl styrene, *MMA* Methyl methacrylate, *BHET* bis-2-hydroxyethyl terephthalate.

^a^Please refer to Fig. 2b for relevant Tc values of respective polymers.

In another example, Chen and coworkers were able to depolymerize PET using simulated sunlight and carbon nanotubes modified with polydopamine (CNT-PDA)^28^. By generating localized heat gradients, the glycolysis reaction in ethylene glycol was promoted forming monomer derivative bis(2-hydroxyethyl) terephthalate (BHET). Hereby, the addition of choline phosphate served to activate ethylene glycol and further promote the transesterification of the PET backbone. This solar thermal catalysis achieved three-fold higher conversion of PET (100%) and yield of BHET (51%) compared to the purely thermal depolymerization at the same temperature. In addition, the light generated local heating gradients also allowed depolymerization at milder temperatures (150 °C) at which a purely thermal approach was not possible (Table 1, entry 2).

Both examples demonstrated advantages, such as higher selectivity and depolymerization efficiency over conventional thermal methods. The main factor contributing to this improvement was the presence of localized temperature gradients, where the temperature near the surface of the light-responsive catalyst was typically higher than in bulk. As a result, the depolymerization near the catalyst proceeded more efficiently despite the bulk temperature being lower. Additionally, the overall concentration of reactive species is reduced through this localized effect leading to less formation of byproducts. Overall, the use of light instead of heat enabled reduction in bulk depolymerization temperatures and selective depolymerization of complex mixtures highlighting its potential as a more sustainable chemical recycling approach.

#### Controlled thermal/photothermal depolymerization

Recently, reactivatable end groups in polymethacrylate polymers generated through RDRP have been exploited to facilitate the conversion back to monomers under milder conditions (Fig. 2d)^16^. While the heat driven depolymerization of methacrylates is generally a known process its waste recycling is predominantly achieved by pyrolysis requiring temperatures of up to 450 °C^29^. RDRP methods have shown a significant reduction in temperature to achieve monomer recovery in high yields. Hereby, the weaker bond strengths between polymer chain and reactivatable end group enable radical initiation at lower temperatures. The thermal depolymerization of polymethacrylates produced by RDRP methods, such as reversible addition-fragmentation chain-transfer (RAFT) polymerization, atom transfer radical polymerization (ATRP), and iodine transfer polymerization (ITP), was recently summarized by Anastasaki and coworkers^16^. Various methacrylate monomers were regenerated from the corresponding polymers in excellent yields up to 92% at temperatures as low as 120 °C. Most examples have so far used RAFT polymerization.

The use of light to drive RDRP/RAFT, either by directly activating the end group (photoiniferter) or by photoinduced electron transfer from a photocatalyst (PET-RAFT) was recently exploited to generate radicals for initiation of the depolymerization equilibrium (Fig. 2di). The use of light and heat to promote controlled depolymerization, known as photothermal depolymerization, offers advantages over conventional thermal depolymerization methods. Photothermal depolymerization typically occurs under milder conditions i.e., at lower temperatures, due to light-induced initiation of the depolymerization equilibrium either by photoiniferter or PET-RAFT mechanisms.

For instance, Sumerlin and coworkers used light as a trigger to directly cleave the C‒S bond between the polymer chain and the RAFT end group i.e., via a photoiniferter mechanism^30^. They depolymerized PMMA (*M*_n_ ∼ 6 kg/mol, *Đ* ∼ 1.2‒1.3) terminated by different RAFT-end groups—trithiocarbonate (TTC), dithiocarbamates (DTC), *para*-substituted dithiobenzoate (DTB)—at 5 mM in dioxane using different wavelengths of light at 100 °C (Table 2, entries 5−7). The depolymerization of most of the end groups studied here proceeded the fastest with UV-light and led to up to 70% monomer recovery within 24 h. From the derived apparent rate constants of depolymerization, all photo-assisted light conditions yielded greater apparent rate constants compared to thermolysis alone.

**Table 2:** Photothermal depolymerization examples of polymethacrylates controlled by RAFT or ATRP methods

Entry	Method	Monomer	Conditions^a^	Light source, temperature	Catalyst	Time	Yield	Refs.
1	RAFT	MMA	5 mM, Dioxane, Dithiobenzoate	Green light, 510 nm (2.3 mW cm^‒2^) 100 °C	Eosin Y (100 ppm)	8 h	82%	^31^
2		MMA	25 mM, Dioxane, Dithiobenzoate	Green light, 510 nm (2.3 mW cm^‒2^), 100 °C	Eosin Y (100 ppm)	6 h	80%	^32^
3		PEGMA	5 mM, Dioxane, Dithiobenzoate	Green light, 510 nm (2.3 mW cm^‒2^), 100 °C	Eosin Y(100 ppm)	8 h	80%	^31^
4		BzMA	5 mM, Dioxane, Dithiobenzoate	Green light, 510 nm (2.3 mW cm^‒2^), 100 °C	Eosin Y(100 ppm)	8 h	78%	^31^
5		MMA	5 mM, Dioxane, Dithiobenzoate	UV light, 365 nm (0.7 mW cm^‒2^), 100 °C	‒	24 h	70%	^30^
6		MMA	5 mM, Dioxane, Trithiocarbonate	Blue light, 453 nm (0.6 mW cm^‒2^) 100 °C	‒	24 h	70%	^30^
7		MMA	5 mM, Dioxane, Dithiocarbamate	UV light, 365 nm (0.7 mW cm^‒2^), 100 °C	‒	24 h	70%	^30^
8		DMAEMA	5 mM, Dioxane, Dithiobenzoate	Green light, 510 nm (2.3 mW cm^‒2^), 100 °C	Eosin Y (100 ppm)	8 h	66%	^31^
9	ATRP	BzMA	50 mM, Dichlorobenzene	Blue light, 460 nm (7.3 mW cm^‒2^) 100 °C	0.05 eq. FeCl_2_, 0.05 eq.TBABr	2 h	59%, (84% at 170 °C, 1 h)	^34^

*MMA* Methyl methacrylate, *BzMA* Benzyl methacrylate, *DMAEMA*  2-(Dimethylamino)ethyl methacrylate, *PEGMA* Ethylene glycol methyl ether methacrylate.

^a^Polymer concentration based on repeat unit, solvent, RAFT end-group (if applicable).

Anastasaki and coworkers applied photocatalyst Eosin Y (100 ppm) to activate the dithiobenzoate RAFT end-group thereby initiating the depolymerization of PMMA under visible light^31^. This study demonstrated that a minimal amount of Eosin Y (100 ppm), in conjunction with green light and heat (100 °C) at 5 mM in dioxane, increased the depolymerization efficiency from 16% to 37% within an hour. Using these conditions PMMA (*M*_n_ ∼ 6.3 kg/mol, *Đ* = 1.14) was depolymerized yielding up to 82% of monomer after 8 h (Table 2, entry 1). The authors also successfully applied these conditions to depolymerize various methacrylate derivatives such as poly(benzyl methacrylate) (PBzMA), poly(2-(dimethylamino)ethyl methacrylate) (PDMAEMA), and poly(ethylene glycol) methyl ether methacrylate (PEGMA) in 78%, 66% and 80%, respectively (Table 2, entries 3‒4, 8). Although this approach successfully used light to initiate the depolymerization, considerable thermal contributions were observed when light was turned off.

Shortly after, Anastasaki and coworkers reported the ability to achieve precise temporal control under visible light irradiation with adjustment of the reaction conditions^32^. This approach ensured controlled depolymerization of the PMMA polymer chains (*M*_n_ ∼ 6.6 kg/mol, *Đ* = 1.11) by using conditions (Table 2, entry 2) that favor the deactivation of the propagating species over uncontrolled depropagation. This light controlled depolymerization method was performed at a lower temperature (90 °C) and higher concentration of PMMA (25 mM) to limit the thermal depolymerization. Increased macro chain transfer agent (macroCTA) concentrations favored deactivation in the RDRP equilibrium and thus enabled the step-by-step depolymerization of monomers. By using these conditions, minimal depolymerization was observed in the absence of light irradiation. The gradual decrease in molecular weight under irradiation was supported by size exclusion chromatography (SEC) traces, while no distinct shift in molecular weights occurred in the dark. In addition, the authors explored the versatility of the system in both dioxane and DMSO and showed that both solvents were equally efficient in imparting temporal control, albeit a slightly lower monomer yield was achieved in DMSO (27%) compared to dioxane (32%). The temporal control over the depolymerization of several polymethacrylates—PBzMA, poly(*n*-butyl methacrylate) (P*n*BMA), PMMA— containing a TTC end-group was demonstrated.

In addition to RAFT polymerization, ATRP stands out as one of the most widely employed techniques for controlled radical polymerization, owing to its versatility in polymerizing monomers with various functional groups^33^. Like RAFT, ATRP has been applied for the thermal depolymerization of polymethacrylates producing monomers in up to 78% yield^16^. Contrary to RAFT, these approaches required catalysts to facilitate the depolymerization i.e., iron or copper chloride, and generally used higher temperatures.

More recently, the Anastasaki group introduced a photocatalytic ATRP method which enabled a reduction in the reaction temperature from 170 to 100 °C as well as photocontrol over the system^34^. The study revealed that only ppm concentrations of iron-based catalysts, coupled with blue light irradiation, were necessary to facilitate monomer recovery (up to 85%) from ATRP-synthesized PBzMA (*M*_n_ ∼ 5‒6.3 kg/mol, *Đ* ∼ 1.2‒1.3, Table 2, entry 9). While higher depolymerization yields were achieved at higher temperatures (170 °C), the thermal contribution was significant. By decreasing the temperature to 100 °C and reducing the catalyst loading to 0.05 eq. (with respect to the polymer chain) light driven temporal control over the depolymerization was achieved. This is owed to a combination of the low catalyst loading with the light-dependent regeneration of the active catalyst species Fe^II^Cl_2_ from Fe^III^Cl_3_ (Fig. 2dii). This approach could be applied at high polymer loading (up to 2 M) and ensured good preservation of end-group fidelity. Compared to previous photothermal depolymerization examples using RAFT polymers, this ATRP approach produced good yields of 59% within an hour compared to >8 h used for RAFT approaches. The ATRP approach is also more robust towards elevated temperatures, as RAFT end groups may undergo decomposition above 150 °C^35^. ATRP’s higher temperature tolerance opens up opportunities to broaden the depolymerization scope to include polymers with higher *T*_c_. While ATRP is most commonly used for the polymerization of (meth)acrylates, styrene, and styrene derivatives, it has also been applied to polymerize other monomers, such as acrylonitrile, 4-vinylpyridine or (meth)acrylamides enabling the possible expansion to a broader set of material classes^36^. Alternatively, ATRP also holds promise for upcycling halogenated materials like PVC, where structural defects present in PVC, i.e., allylic chloride or tertiary C‒Cl groups, can act as initiating sites^37^.

### Photocatalytic degradation to small molecules

#### Proton coupled electron transfer

**Fig. 3:**
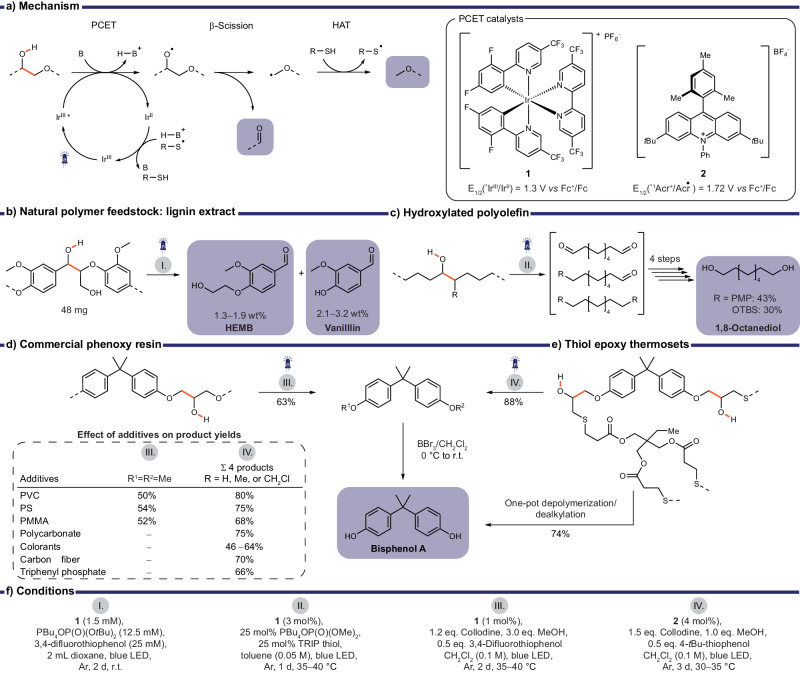
Proton-coupled electron transfer to break down hydroxy containing polymers. **a** PCET mechanism resulting in cleavage of orange bonds, structures of respective photocatalysts. Chemical recycling of (**b**) natural polymer feedstocks like lignin extracts, (**c**) hydroxylated polyolefins, (**d**) a commercial phenoxy resin, (**e**) thiol epoxy thermosets. **f** Reaction conditions of respective methods.

The selective cleavage of polymer backbones to generate defined degradation products is highly desirable and very challenging due to the inert C‒C backbone of most commodity polymers. A recent photoredox strategy, proton coupled electron transfer (PCET), enabled the selective activation of hydroxy groups which are attached to the polymer backbone resulting in directed C‒C bond cleavage (Fig. 3a)^38–42^. The O‒H bond is homolytically cleaved through a concerted electron transfer to an excited state oxidant (**1** or **2**, Fig. 3a), while a weak Brønsted base abstracts the proton. The generated alkoxy radical promptly undergoes *β*-scission resulting in a carbonyl and a more stable alkyl-radical which is protonated by a thiol, acting as a HAT catalyst. The selective cleavage of a C‒C bond vicinal to a hydroxy group was favored when the generated C-centered radical was stabilized e. g. by an ether or benzyl moiety^41^. Choice of base and catalyst needed to be closely matched to the nature of the substrate. PCET was successful when the effective bond dissociation free energy (BDFE) – calculated from the substrate’s redox potential and the base p*K*_a_– exceeded that of the substrate’s O‒H bond (BDFE 105 kcal/mol)^38^. This approach was successful in degrading different types of hydroxy containing polymers into well-defined products, such as vanillin, 4-(2-hydroxyethoxy)-3-methoxybenzaldehyde (HEMB), octane diol or bisphenol A precursors (Fig. 3b–f)^40–42^.

The selectivity of this method enabled the conversion of lignin, a natural polymer found in wood, to small organic building blocks. Though not plastic waste, this transformation may be valuable to upcycle lignin extracted from timber waste and is an exciting development for the production of aromatics from sustainable resources. Various lignin depolymerization processes, i.e., using heat, acid/base, or redox chemistry have been explored producing a variety of substituted phenolic compounds^43^. After further modification these depolymerization products may be used as polymer building blocks e.g., terephthalic acid as a precursor of PET or specific alkanes or alcohols as fuels. The highly complex structure of lignin makes selective product formation challenging. Its structure consists of a variety of benzylic building blocks linked by C‒C and C‒O bonds with the most common link in the backbone being a *β*-O-4 moiety^44^. Model compounds of this characteristic link have been widely studied and selectively cleaved using photoredox catalysis^39,40,45–53^. Recently, PCET was used to depolymerize native lignin which generated various small molecules such as HEMB from 1.3 to 1.9 wt% and vanillin from 2.1 to 3.2 wt% depending on the natural lignin source (Fig. 3b)^40^.

The PCET approach was also applied to a polymer without any heteroatoms in the backbone which increases the difficulty of selective degradation^41^. The incorporation a hydroxy group in α-position to either silylether- (OTBS-) or *p*-methoxyphenyl- (PMP-) functionalized C‒C backbone enabled selective backbone scission under mild reaction conditions (Fig. 3c). After PCET a mixture of the three expected products was obtained in 26% or 85%, for OTBS- or PMP-derivatives respectively, which could be transformed into a single product, 1,8-octanediol, in four steps in overall yields of 30% or 43% respectively. Though most commodity plastics do not contain hydroxy groups, recent advances in polymer post modification enabled introduction of carbonyl or hydroxy groups along the backbone^54,55^. Combined with these efforts the PCET methodology may give access to selective degradation of more widely used plastics in the future.

However, two examples of commercially available epoxy resins with similar backbone structures were successfully converted into bisphenol A precursors under mild conditions in good yields (63%, see Fig. 3d, 88%: sum of 4 products, see Fig. 3e)^41,42^. In both examples PCET was expected to form a phenoxy acetaldehyde which was found to be unstable and eliminated CO resulting in methoxy-benzene containing products. To improve the selective formation of one product, methanol was added which reacted with the unstable acetaldehyde intermediate and subsequently underwent a second PCET reaction sequence to give bisphenol A precursor (63% for phenoxy resin). A consecutive dealkylation of this precursor provided bisphenol A, a monomer/precursor/additive used in a variety of polymerization processes. For thiol epoxy thermosets this was accomplished in one-pot with a yield of 74% despite the initial polymer not being soluble, though the material was mechanically ground to a powder pre-depolymerization^42^.

The extraordinary selectivity of this methodology was demonstrated by adding equal masses of other commodity polymers (PVC, PS, PMMA, polycarbonate) to the depolymerization process^41,42^. The added polymers were predominantly unaffected, proven by minimal molecular weight changes in SEC, and the depolymerization yields were only slightly diminished. Even the addition of colorants and other additives had a negligible effect on the depolymerization of thiol epoxy thermosets^42^. The presence of additives is generally considered an immense challenge for plastics recycling, making the resilience of this approach an exciting advancement. The depolymerization was applicable to thermosets with different mechanical properties (varying crosslinking densities) though the glass transition temperatures (*T*_g_) of around 30 °C and the decomposition temperatures (*T*_d,5%_) above 300 °C remained consistent. Its ability to convert initially insoluble resins to valuable small molecules under mild conditions holds promise for future catalytic approaches to process mostly insoluble commodity polymers.

#### LMCT induced β-scission of inactivated alcohols

Contrary to photoredox catalysis where an excited state reductant or oxidant may participate in an intermolecular SET to a substrate, LMCT requires the coordination of the reactant to the metal center^56^. LMCT may be simplified as a light-induced homolytic bond cleavage between metal M and a ligand Z in a complex (M^n^L_n_Z) to form reduced metal center (M^n‒1^L_n_) and radical Z^•^. This strategy was used by Soo and coworkers to selectively cleave C‒C bonds of inactivated alcohols under visible light irradiation by oxygen coordination to a vanadium photocatalyst^57^. In aerobic conditions the LMCT-produced-radical formed oxygenated products, such as aldehydes and alcohols, whereby 1,4-cyclohexadiene served as an additive to avoid overoxidation.

The authors proposed that initial LMCT led to β-scission forming an alkyl radical and catalyst**-**coordinated formaldehyde. Subsequent reactions with O_2_ were thought to produce formic acid and carbon dioxide as oxidation products as well as a metal coordinated peroxide intermediate. Final homolytic cleavage of the peroxide in combination with proton transfers produced a hydroxylated polymer of shorter chain length and water, upon which the cycle is repeated.

Various polymers containing oxygen moieties able to coordinate to the catalyst could be degraded successfully (>95% conversion) under visible light irradiation in air in absence of the additive. Most examples proceeded at room temperature (r.t.) but higher temperatures (85 °C) were required to dissolve PE containing polymers. Mono-alcohol functionalized PE produced 5% formic acid in 6 d and an unquantified amount of CO_2_. The volatility of formic acid was also suggested to contribute to the reduced yield.

Higher yields and selectivity towards formic acid/methyl formate (70‒75%) were achieved for polymers containing additional oxygen moieties in the backbone, e.g., polyethylene glycol (PEG) or polycaprolactone-PEG block polymer (PCL-PEG-PCL). Though mechanistically different to PCET this approach also results in β-scission of the C‒C backbone. In contrast to PCET, aerobic conditions were required to regenerate hydroxy-groups after chain-scission which were then able to coordinate to the catalyst again. Aerobic conditions facilitated the cleavage of the C‒C backbone, but also caused overoxidation to less desirable CO_2_. This example demonstrates the intricate equilibrium required between maintaining reaction conditions that are robust enough to degrade C‒C bonds while avoiding the unwanted transformation into less desirable products, such as CO_2_. This approach remains limited to hydroxy terminated polymers required for the coordination to the catalyst.

#### HAT induced oxidative cleavage in the presence of O_2_

**Fig. 4:**
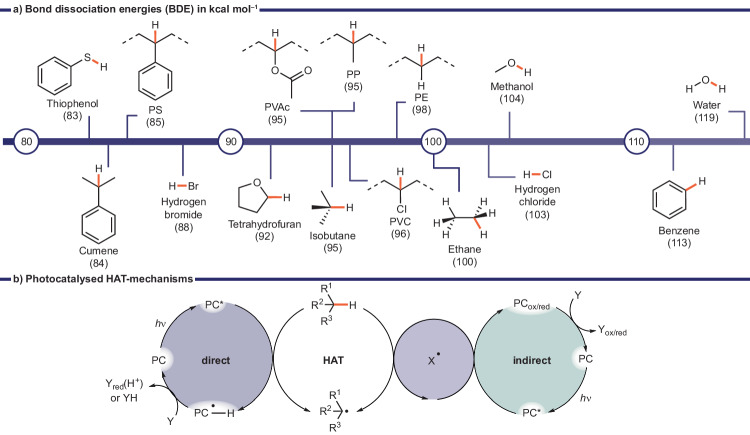
Hydrogen atom transfer (HAT). **a** Bond dissociation energies (BDE in kcal mol^‒1^ referring to bonds in orange) of select compounds^58,117^ and commodity polymers^61^. **b** Photocatalyzed direct and indirect HAT mechanisms^118^.

Most commercially employed polymers only contain C‒C backbones that lack functional groups such as esters or amides, which may easily be cleaved e.g., by solvolysis. Recent advancements have enabled the selective activation of otherwise unreactive C‒H (sp^3^) bonds through the application of photocatalysts^58^. When a photocatalyst is excited, it can facilitate a concerted hydrogen abstraction (H-radical), whereby labile C‒H bonds are more prone to homolytic cleavage. Consequently, the bond dissociation energy (BDE) of C‒H bonds may be used as an indicator of reactivity. The BDEs of select structures, including the most labile C‒H bonds of commodity polymers, are highlighted in Fig. 4a. Aside from BDEs many other factors may affect the C‒H bond cleavage such as the stability of the generated radical species, hyperconjugation, steric effects, and the electrophilic/nucleophilic character of the C‒H bond based on neighboring heteroatoms (N, O). Hydrogen atom transfers may occur directly by an excited state PC or indirectly by a reactive species (X^•^) which is generated by a photocatalyst (Fig. 4b).

**Fig. 5:**
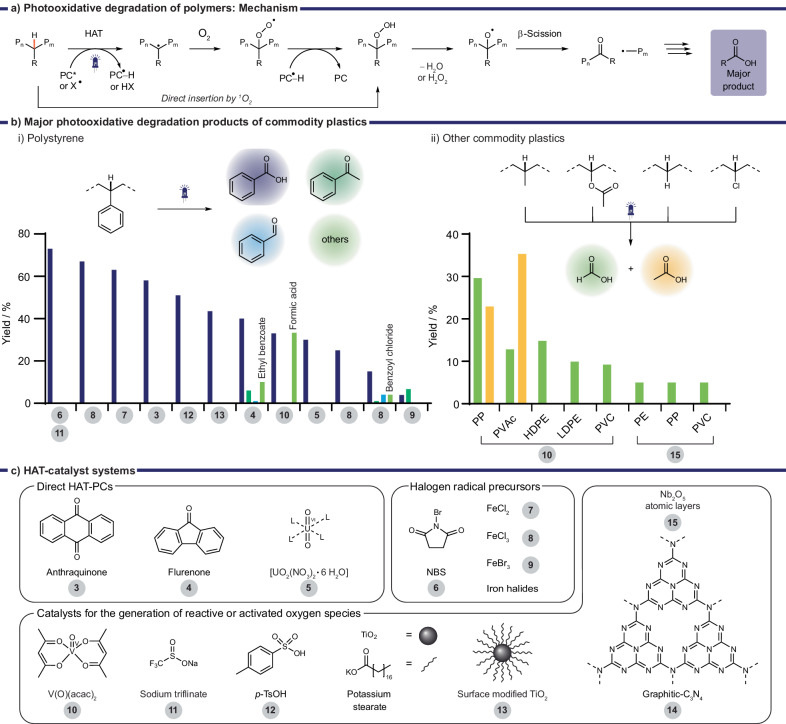
Photooxidative degradation of C‒C polymers. **a** Mechanism^63,64^, (**b**) Major products obtained for PS (i) and other commodity plastics (ii). **c** Photocatalyst structures **3**‒**15** used for direct HAT, halogen radical generation, and generation of reactive or activated oxygen species.

Most synthetic strategies use HAT to selectively functionalize unreactive C‒H bonds and can introduce a variety of functional groups to create value-added materials^59,60^. This approach has also been applied to cleave C‒C polymer backbones by using oxygen, reactive or activated oxygen species as oxidants to generate value added small molecules. Hereby, initial HAT generates a carbon centered radical which subsequently reacts with oxygen, reactive or activated oxygen species (denoted as [O]) forming an alkoxy radical (Fig. 5a). This oxygen centered radical species then undergoes β-scission, cleaving the polymer backbone, and generating a carbonyl group and an alkyl radical. Both fragments undergo further HAT and oxidation steps which generate small molecules. In most examples discussed in this review the major products of this process are carboxylic acids with a residue related to the initial polymer side chain, or backbone. For example, PS degradation predominantly led to formation of benzoic acid (Fig. 5bi) whereas PE and PP both led to formation of formic acid and PP additionally produced acetic acid (Fig. 5bii). Though this process does not regenerate monomers, the produced small molecules may be used in other relevant industrial applications and their potential value and uses are discussed in the conclusion.

Most examples using HAT initiated oxidative photodegradation were reported on PS due to its low C‒H BDE (85 kcal mol^‒1^ *vs* 98 kcal mol^‒1^ of PE)^61^ and its good solubility in organic solvents, which facilitates chemical processing. The tertiary (3°) C‒H bond in the backbone is easily cleaved by HAT-catalysts which initiates the photooxidative degradation. The radical-stabilizing benzyl group may also have a favorable effect on the subsequent radical fragmentation pathways predominantly generating benzoic acid in up to 73% yield (Table 3)^62^. Reported examples can be divided predominantly into three main categories based on the reactive species responsible for the initial HAT step (Fig. 5c): carbonyl containing HAT photocatalysts (**3**‒**5**)^63,64^, light generated halogen radicals (**6**‒**9**)^65–68^, and light driven generation of reactive oxygen species (**10**‒**15**)^61,62,69–71^. It should be noted that so far only reactive or activated oxygen species have successfully converted C‒C polymers other than PS to valuable small molecules.

**Table 3:** Reported examples of photooxidative degradation of PS sorted by decreasing yields of the major product

#	Light source, *T*	Conditions	Catalyst	Product	Time	Yield	Refs.
1	390 nm (0.23 W cm^‒2^), r.t.	EtOAc (0.1 M), 50 mol% Sodium triflinate, O_2_ balloon	NBS(15 mol%)	Benzoic acid (& derivatives by post-functionalization)	16 h	73%^a^ (21‒60%)^b^	^62^
2	390 nm (100 W), r.t.	Acetone (0.1 M), 10 mol% TBACl, 20 mol% Cl_3_CCH_2_OH, O_2_ balloon	FeCl_3_ (10 mol%)	Benzoic acid	5 d	67%^b^	^65^
3	400 nm (20 W), r.t.	CH_2_Cl_2_/MeCN or acetone (0.25 M), O_2_ balloon	FeCl_2_ (2 mol%)	Benzoic acid	66 h	63%^b^	^66^
4	390 nm (52 W), r.t.	MeCN/CH_2_Cl_2_ (1:1, 50 mg/mL), Air	Anthraquinone (5 mol%)	Benzoic acid (formic acid, acetophenone)	48 h	58%^b^ (Large scale: 24%^b^, 59%^a^, 5%^b^)	^64^
5	405 nm (9 W), r.t.	Benzene/CH_3_CN, (1/1 = 52 mg/mL), O_2_ (1 bar)	*p*-TsOH (5 mol%)	Benzoic acid, (Formic acid, Acetophenone)	15 h	51%^b^	^69^
6	370 nm (40 W), r.t.	CH_2_Cl_2_ (0.1 M), O_2_ (1 bar)	Modified TiO_2_ (potassium stearate; 10 mg/mL)	Benzoic acid (& others)	4 h	43.5%^c^ (1%)^c^	^71^
7	450 nm (14.4 W), 50 °C	EtOAc (0.1 M), 1 eq. H_2_SO_4,_ O_2_ balloon	Fluorenone (0.2 eq.)	Benzoic acid (Ethyl benzoate, Acetophenone, Benzaldehyde)	72 h	∼ 40%^d^ (∼ 10%, ∼ 6%, ∼ 1%)^d^	^63^
8	2 x White LEDs (50 W), r.t.	CH_2_Cl_2_ (21 mM), O_2_ atmosphere	V(O)(acac)_2_ (2 mol%)	Formic acid Benzoic acid	4−7 d	33.3%^b^ 33.0%^b^	^61^
9	460 nm (9 W), 40 °C	CH_2_Cl_2_ (0.48 M), 0.2 eq. HCl, O_2_ balloon	[UO_2_(NO_3_)_2_ 6 • H_2_O] (5 mol%)	Benzoic acid	72 h	30%^b^	^72^
10	456 nm (50 W), r.t.	Acetone (40−80 mg/mL), O_2_ balloon	FeCl_3_ (10 mol%)	Benzoic acid	20 h	25%^c^	^68^
11	White (60 W), r.t.	Acetone (80 mg/mL), O_2_	FeCl_3_ (10 mol%)	Benzoic acid (Benzaldehyde, Benzoyl chloride, Acetophenone)	20 h	15%^c^ (4%, 4%, 1%)^c^	^67^
12	456 nm (50 W), r.t.	Acetone (40-80 mg/mL), O_2_ balloon	FeBr_3_ (1.7 mol%) 2.7 mol% LiBr,	Acetophenone Benzoic acid	48 h	6.7%^c^ 3.9%^c^	^68^
13	Xenon (300 W). 150 °C	MeCN (0.3 mg/mL), O_2_ (20 bar)	graphitic-C_3_N_4_ (5 mg/mL)	Benzaldehyde, acetophenone, benzoic acid	8 h cycle x 20	‒	^70^

^a^Yields based on a single repeat unit and calculated by NMR.

^b^Isolated yield.

^c^Determined by gas chromatography (GC).

^d^Determined by high-performance liquid chromatography (HPLC).

Direct HAT by organic photocatalysts followed by oxidative degradation produced benzoic acid from PS in up to 58%^63,64^. Reisner and coworkers used fluorenone as the HAT-photocatalyst in combination with stoichiometric amounts of sulfuric acid in an oxygen atmosphere under visible light irradiation (450 nm, 48 h at 50 °C, see Table 3, entry 7)^63^. Initial HAT followed by oxidation with oxygen produced benzoic acid in ∼40% and ∼20% of other aromatic small molecules such as ethyl benzoate (∼10%), acetophenone (∼6%), and benzaldehyde (∼1%). The formation of 21% CO and 7.5% CO_2_ were also detected which are side products of the oxidative radical fragmentation pathways. Acid addition was required for the transformation, hypothesized to facilitate the elimination of water as well as reduce the activation barrier for β-scission in the oxidative fragmentation pathways (see Fig. 5a). This strategy was applied successfully to commercial PS foam (M_w_ ∼ 250 kg/mol, PDI ∼ 4.2) on gram-scale, resulting in similar yields with an extended reaction time of 72 h.

Shortly after Kokotos and coworkers demonstrated degradation of PS based resins (e.g., poly(styrene-co-divinylbenzene)) to benzoic acid (28%) without acid using near-UV light (390 nm) and anthraquinone as HAT-photocatalyst in air (Table 3, entry 4)^64^. Aside from acting as a HAT photocatalyst, anthraquinone was proposed to generate singlet oxygen which may act as HAT agent or directly insert into the α-benzylic C‒H bond promoting further oxidative degradation (Fig. 5a). Several different PS products, e.g., plastic cups, were degraded to benzoic acid in small scale reactions where transparent products gave yields up to 59%. Colored products gave slightly reduced yields (27%) due to light attenuation by the dyes present in the plastics. The composition of commercial products was, however, not analyzed, and the presence of other additives is likely to affect the yield. The use of benzoic acid as a pharmaceutical drug precursor was also highlighted by synthesizing acetylsalicylic acid commonly known as Aspirin.

Inorganic compounds, such as the uranyl cation UO_2_^2+^, have also shown promising HAT catalyst activity^58^. Jiang and coworkers used [UO_2_(NO_3_)_2 _  • 6 H_2_O] to degrade PS to benzoic acid in 30% yield. They used blue light irradiation in CH_2_Cl_2_ with 0.2. eq. hydrochloric acid in an oxygen atmosphere (Table 3, entry 9). Other polymers with C‒X backbones, e.g., polyether, polycarbonate, or polyesters, could be converted to monomers in moderate to good yields (25‒88%)^72^.

Several examples have shown the successful use of iron chloride salts as HAT catalysts for the photooxidative degradation of PS to predominantly benzoic acid (up to 67%)^65–68^. Both FeCl_2_ and FeCl_3_ were used which produce Cl^•^, the HAT agent, upon irradiation via LMCT. PS of high molecular weight (M_w_ ∼ 230 kg/mol, 67%, Table 3, entry 2^65^; M_w_ ∼ 145 kg/mol: 63%, Table 3, entry 3^68^) as well as various commercial products were successfully degraded with these approaches^65–67^. The use of milder reaction conditions (visible light, air) produced benzaldehyde as the major product in addition to benzoic acid, some benzyl chloride and traces of other benzyl products^67^. However, when an oxygen atmosphere was used, or reaction times were prolonged the aldehyde products were further oxidized to form benzoic acid. Other oxidation products, such as formic acid, were also detected in some cases but not isolated. The use of white or blue light, instead of near-UV light (∼400 nm) appeared to generally decrease the product yield 15‒25% (Table 3, entries 10‒11) *vs* 63‒67% (Table 3, entries 2‒3). This may indicate that ∼400 nm irradiation in the presence of oxygen enhances the oxidative radical fragmentation to yield small molecule products like benzoic acid. However, the use of more selective visible light may be advantageous for the degradation of commercial PS containing fillers, radical scavengers, or UV-absorbers which may hinder the degradation process.

**Fig. 6:**
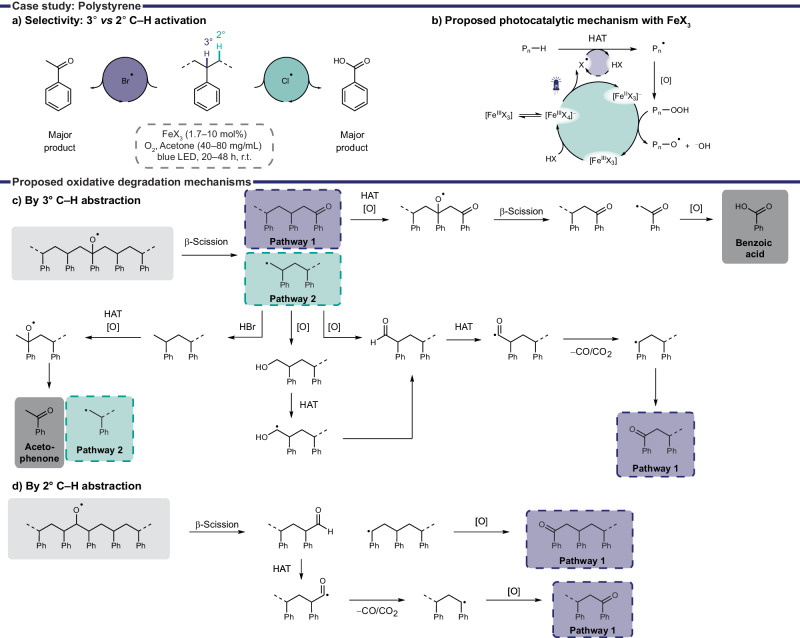
Photooxidative degradation of PS with FeX_3_^68^. **a** Halogen radical dependent selectivity for 2° or 3 °C‒H activation. **b** Proposed photocatalytic mechanism of FeX_3_. **c**, **d** Proposed oxidative degradation pathways generating benzoic acid and acetophenone.

In a recent study Stache and coworkers showed the product selectivity of the PS degradation could be influenced by choice of the generated halogen radical^68^. Br^•^ produced acetophenone as the major product whereas Cl^•^ produced benzoic acid as the major product in an oxygen atmosphere under blue light irradiation (Fig. 6a, Table 3, entries 10, 12**)**. The employed iron catalysts Fe^III^X_3_ were proposed to initially disproportionate to [Fe^III^X_4_]^‒^, and then undergo LMCT generating X^•^ and [Fe^II^X_3_]^‒^ (Fig. 6b). The blue light generated halogen radicals abstract a hydrogen from the PS backbone forming a carbon centered polymer radical P_n_^•^ and HX. Further oxidation with oxygen is proposed to produce a hydroperoxyl intermediate P_n_‒OOH which then oxidizes the reduced iron catalyst [Fe^II^X_3_]^‒^ to Fe^III^X_3_ generating alkoxy radical P_n_‒O^•^. Reaction of the catalyst with HX, produced by the HAT step, closes the photocatalytic cycle.

The halogen dependent product selectivity was explained by a detailed analysis of the C‒H abstraction and following radical fragmentation and oxidation pathways. Proposed intermediates and the product selectivity were supported by subjecting mostly small molecule analogs to the reaction conditions. The study showed that Br^•^ more selectively reacted with the weaker 3° C‒H bond (Fig. 6c) while HAT by Cl^•^ was less selective and cleaved the stronger 2° C‒H bond (Fig. 6d).

Following HAT and oxidation, β-scission of the alkoxy radical breaks the C‒C backbone according to the proposed mechanism (Fig. 6c, d). A carbonyl intermediate and an alkyl radical are produced which are proposed to fragment further in two main pathways. Pathway 1 predominantly produces benzoic acid and is the only pathway accessed by 2° C‒H abstraction (Fig. 6d). However, 3° C‒H abstraction facilitates both pathways 1 and 2 (Fig. 6c). In pathway 2 the alkyl radical is either protonated by HBr leading to formation of acetophenone or reacts with an oxygen species leading to intermediates which equal the starting point of pathway 1. The preferred activation of 3° C‒H by Br^•^ as well as protonation of the methyl radical in pathway 2 by HBr explain the preferred formation of acetophenone over benzoic acid. Overall, the oxidative degradation of PS with FeBr_3_ and catalytic amounts of LiBr produced acetophenone in 6.7% and benzoic acid in 3.9% under blue light irradiation in an oxygen atmosphere in 48 h (Table 3, entry 10). The same conditions but using FeCl_3_ and less time (20 h) primarily produced benzoic acid in 25% (Table 3, entry 12). This example highlights the importance of understanding the mechanism of fragmentation pathways to increase selectivity but also demonstrates their complexity.

Liu and coworkers reported the use of AlCl_3_ to efficiently degrade PS to predominantly benzene using UV light (254 nm) in an argon atmosphere followed by conversion to predominantly diphenylmethane^73^. Though the mechanism was not discussed, it is mentioned here due to the catalyst’s structural similarity to iron halide salts. However, the use of an argon atmosphere makes it extremely unlikely for the mechanism to proceed in analogy to previously discussed iron halide examples which required the presence of oxygen. Regardless, this approach shares some similarities with the established industrial process for diphenylmethane production using Friedel-Crafts alkylation. In addition, its economic viability, as determined through a techno-economic analysis, makes it a highly promising approach.

The UV-light induced photooxidative degradation of polymers driven by the presence of reactive oxygen species has been long known and often leads to deterioration of material properties^74,75^. In more recent developments, researchers have leveraged oxygen-initiated HAT to intentionally degrade polymers with C‒C backbones, ultimately yielding specific and valuable small molecule products^61,62,69–71^.

McInnes, Qi, Xiao and coworkers demonstrated that using catalytic amounts of various acids in an oxygen atmosphere (1 bar) under 405 nm irradiation could degrade PS (M_w_ ∼ 172 kg/mol) to benzoic acid, formic acid and acetophenone (Table 3, entry 5)^69^. Using 5 mol% triflic acid in an acetonitrile benzene mixture (1:1), benzoic acid was isolated in 51%. A similar amount of formic acid and a negligible amount of acetophenone were detected by ^1^H NMR. A PS-acid adduct, which absorbs at the applied wavelength, is proposed to generate singlet oxygen by an excited state energy transfer. Singlet oxygen in turn acts as the hydrogen atom abstractor or directly inserts into the C‒H bond as previously suggested (see Fig. 5) which initiates further oxidative degradation. This method’s applicability to commercial products, its demonstrated upscaling potential in a photoflow reactor and use of cost-effective reagents show substantial potential for future industrial applications. Though further solvent optimization may be required to offer a more environmentally friendly alternative to the use of benzene.

A dual HAT catalyst system (*N*-bromosuccinimide (NBS), sodium triflinate, Table 3, entry 1) was used by Das and coworkers to degrade PS to predominantly benzoic acid in good yields up to 73% in 16 h using 390 nm light^62^. Light-induced generation of reactive HAT agents Br^•^, a succinimide radical and oxygen activated pentacoordinate sulfide CF_3_SO_4_^‒^ were proposed to initiate the oxidative photodegradation. The method was successful for a variety of molecular weights (M_w_ ∼ 260‒546 kg/mol), dispersities and several commercial products on gram scales though large amounts of NBS (15 mol%) and sodium triflinate (50 mol%) were required. As a proof of concept, the obtained benzoic acid was used to synthesize drug derivatives or other commonly used chemicals, e.g., benzene or toluene, to highlight its potential for substituting petrochemically derived products.

While several heterogeneous catalysts have been used to degrade plastics the focus rarely was on the selective generation of small molecules^17,18,76–78^. Heterogeneous catalysts, such as semiconductors, offer advantages for industrial applications due to their robustness and facile recovery. Semiconductors consist of a valence band (VB) and a conduction band (CB) which are separated by a band gap. Upon irradiation with light that is equal to or higher than the energy of the band gap, an electron is excited from the VB to the CB and entails light induced redox activity.

Ma, Weng and coworkers used a graphitic carbon nitride catalyst (g-C_3_N_4_) to degrade PS (M_w_ ∼ 0.8‒110 kg/mol) to benzaldehyde, acetophenone, and benzoic acid using broad spectrum light and elevated temperatures (150 °C) in a pressurized oxygen atmosphere (20 bar, Table 3, entry 13)^70^. Visible light irradiation equivalent or higher in energy than that of the bandgap of this semiconductor (2.7 eV) resulted in excitation of an electron from the VB (1.4 V *vs* NHE at pH 7) to the CB (‒1.3 V vs NHE at pH 7)^79^. According to the reported mechanism, the electron in the CB then reduced O_2_ (E^0^(O_2_/^•^O_2_^‒^) = ‒0.16 V) to the reactive oxygen species ^•^O_2_^‒^ which acted as the HAT agent^70,79^. Values for the conversion and selectivity were calculated based on a product’s carbon content and are hence not used to compare to other studies^70^. Elevated temperatures increased the conversion but, similar to longer reaction times, also increased the overoxidation to CO_2_ or CO. The product selectivity could therefore be varied by adjusting the mass flow rate in a reactor setup at a fixed catalyst loading: faster flow rates largely resulted in benzaldehyde and smaller amounts of acetophenone; slower rates predominantly resulted in benzoic acid. Other heterogeneous catalysts tested in this study, e.g., TiO_2_ showed less selectivity for small organic molecules and increased CO_2_ formation.

More recently TiO_2_ particles modified with organic residues such as potassium stearate (**13**, Fig. 5c), enabled the oxidative degradation of PS at ambient temperature and pressure to 43.5% benzoic acid in 4 h (Table 3, entry 6)^71^. UV-excitation of the modified TiO_2_ catalyst was proposed to generate reactive oxygen species (^•^O_2_^‒^, ^1^O_2_) which initiated the PS degradation via HAT as previously discussed. Surprisingly, no CO_2_ formation was detected which was attributed to the milder reaction conditions.

Simulated natural environment conditions—broad spectrum light, water, ambient temperature, and air—were used by Xie and coworkers to degrade PE, PP and PVC (in powder form) to CO_2_ and acetic acid with single layer Nb_2_O_5_ (39.5‒47.4 µg g_cat_^‒1^ h^‒1^)^80^. The authors proposed the initial photodegradation of the plastics to CO_2_ via ^•^OH and O_2_ mediated C‒C bond cleavage. According to the proposed mechanism, photoexcited holes in the VB ( + 2.5 V *vs* NHE, pH 7) oxidized H_2_O to ^•^OH radicals and electrons in the CB (‒0.9 V *vs* NHE, pH 7) stepwise reduced O_2_ to H_2_O. The generated CO_2_ was subsequently reduced to acetic acid by the same photocatalyst via C‒C coupling of ^•^COOH intermediates accompanied by the oxidation of H_2_O to O_2_. Using this method PE was completely degraded to CO_2_ within 40 hours. However, the yield of acetic acid remained low (39.5‒47.4 µg g_cat_^‒1^ h^‒1^) and requires significant catalyst improvement to achieve efficient conversion of CO_2_ to valuable small molecules, such as acetic acid.

A recent study by Soo and coworkers reported the successful oxidative degradation of several commodity plastics aside from PS by using commercially available catalyst V(O)(acac)_2_ in an oxygen atmosphere under white light irradiation^61^. Oxygen was proposed to coordinate to the catalyst thereby oxidizing V^IV^(O)(acac)_2_ to V^V^. Light induced LMCT then resulted in C‒H oxidation by the catalyst coordinated oxygen and subsequent C‒C cleavage reactions. Carboxylic acids were predominantly isolated, and CO_2_ was identified as undesired overoxidation product which was increasingly formed at prolonged reaction times. A large variety of conventional plastics, such as PS (Table 3, entry 8), PP, PVAc, HDPE, LDPE, and PVC, were successfully degraded using this method and produced formic acid and acetic acid in combined yields of 9‒66%. The robustness of the method was demonstrated by successfully degrading commercial multilayer packaging (e.g., PP-EVOH-PP) or Styrofoam contaminated with an equal amount of organic matter, such as canola oil. Larger scale reactions on gram scale were also successful using a photo flow reactor.

A notable challenge for the conversion of C‒C commodity polymers like HDPE, LDPE or PP is their limited solubility. This was overcome by heating plastic-dichloromethane mixtures to 110 °C in an autoclave which gave well dispersed plastic colloidal suspensions. Longer reaction times of up to 7 days were also necessary to improve yields whereby further extension of the reaction time led to increased CO_2_ formation, yet again highlighting the delicate balance between yield and selectivity. The selective C‒C cleavage of PVC is particularly challenging due to the more labile C‒Cl bond^11^. The limited success of this method to degrade PVC is evident from the particularly low yield of formic acid which was attributed to the deactivation of the catalyst by in situ formed HCl^61^.

Successfully converting various C‒C commodity plastics, including mixtures and contaminants, into useful small molecules is a promising advancement. However, further catalyst development is needed to improve yields, selectivity and reduce reaction times.

#### Side chain reactivity for backbone cleavage

**Fig. 7:**
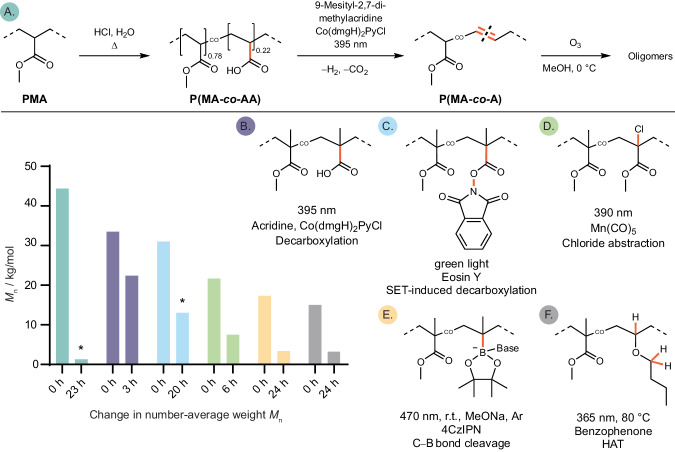
Structures of copolymers with corresponding reaction conditions for photodegradation (A‒F). The most reactive bonds used to initiate degradation, predominantly by radical mechanisms (B‒F), are highlighted in orange. Initial *M*_n_ are compared to *M*_n_ after photodegradation (left)^81–86^. ^*^ Reported as *M*_p_.

Several recently reported examples use photocatalysts to initiate degradation of C‒C polymer backbones by cleaving reactive side chains^81–86^. These approaches avoid the introduction of weak links in the polymer backbone by copolymerization, a strategy to introduce degradability, which can lead to depreciated thermomechanical properties^87,88^. The desired reactivity was introduced by choice of sidechains facilitating either decarboxylation^81,83^, SET induced decarboxylation^82^, Cl-abstraction^84^, C‒B bond cleavage^86^, or HAT^85^ to initiate the decomposition process (Fig. 7).

The largest change in molecular weight was reported by Sumerlin and coworkers who, in the first step, were able to convert poly(methyl acrylate) (PMA) to methylacrylate-acrylic acid copolymers (P(MA-*co*-AA)) by partial hydrolysis of MA to AA^83^. Photocatalytic decarboxylation of P(MA-*co*-AA) produced alkene copolymer P(MA-*co*-A) which was then cleaved by ozonolysis yielding oligomers of drastically lower molecular weight compared to other methods (Fig. 7A*vs* B‒F)^81,83–85^. In contrast to the examples B‒F where the polymer structure is designed to make it degradable, this approach (example A, Fig. 7) is generally applicable to acrylate polymers which are otherwise not easily depolymerized (unlike PMMA)^83^. All of these approaches (A‒F, Fig. 7) produced polymers of shorter chain lengths which require further property analysis to identify their potential uses. Alternatively, further chemical transformations are required to give useful products. While oligomers are typically considered undesirable byproducts in degradation processes, some examples derived from PE or PP, have proven useful as surfactants, waxes, or lubricants, significantly enhancing product value^89–91^.

#### Photoreforming with heterogeneous photoredox catalysts

Plastics may also be used as feedstock to generate fuel^92^. Photoreforming applies semiconductors to generate H_2_ from an aqueous solvent coupled with a biomass oxidant reaction. Hereby, excitation of electrons from the VB to the CB allows reduction of H_2_O to H_2_ if the CB of the semiconductor is more negative than the reduction potential of H^+^/H_2_^93^. In turn, the generated holes in the VB facilitate the oxidation of organic matter if the VB is more positive than the oxidation potential of the substrate. As such organic waste may serve as sustainable sacrificial electron donor to overcome the energy intense oxidation of H_2_O to O_2_ which is the limiting factor of water splitting. As a byproduct, the applied organic matter, such as biobased waste polymers (e.g., lignin, sugars) or plastic waste, is degraded to CO_2_, H_2_O or in some cases also valuable small molecules^20,94^.

In the context of plastic waste, the majority of examples used pretreated PET or polylactic acid (PLA), meaning the polymer is hydrolyzed to small molecules prior to photoreforming^20,93^. Hydrogen production rates of up to 63 mmol_H2_ g_cat_^‒1^ h^‒1^ were achieved for the photoreforming of PLA using CdS/CdO_x_ quantum dots in alkaline aqueous solution^20,95^. However, photoreforming of C‒C backbone plastics is more challenging due to their chemical inertness and impaired solubility. More soluble components are more easily converted due to their facile interaction with the surface of heterogeneous catalysts whose performance is generally improved with greater surface area^96^. The first photoreforming reports of PE and PVC powders achieved relatively low rates of 0.031 and 0.015 mmol_H2_ g_cat_^‒1^ h^‒1^ respectively using 5% Pt/TiO_2_ in 5 M NaOH^97^. More recent examples achieved higher rates of 1.7 mmol_H2_ g_cat_^‒1^ h^‒1^ for PE microplastic with an Ag_2_/Fe-metal organic framework (MOF) catalyst, and 0.60 to 0.65 mmol_H2_ g_cat_^‒1^ h^‒1^ (for PP and PE powders) using a Co-Ga_2_O_3_ catalyst^98,99^. Other examples required initial chemical modification or degradation of PE or PP through plasma treatment or heating in the presence of strong acids achieving 0.10 mmol_H2_ g_cat_^‒1^ h^‒1^ with commercial TiO_2_ catalyst and 1.1 mmol_H2_ g_cat_^‒1^ h^‒1^ with MoS_2_-tipped CdS nanorods^100,101^. Generally, the selective formation of monomers or small molecules over fuel is more valuable from a circular economy perspective. However, photoreforming may offer a valuable strategy to transform contaminated or difficult to recycle plastic waste to H_2_ to fulfill a growing demand for green fuels^102,103^.

In conclusion, light offers a sustainable approach for converting polymers either to monomers or to other small molecules by selectively activating decomposition pathways thereby reducing the energy input otherwise required for thermal processes. Light-induced depolymerization back to monomer either enabled the reduction of applied heat or used light to produce heat which overall increased the energy efficiency. Compared to heat-based depolymerization, the formation of side-products was reduced, and polymer mixtures of drastically different *T*_c_ could selectively be depolymerized circumventing tedious preprocessing. This approach is still constrained to polymers with low *T*_c_ which limits the application to most used commodity polymers (e.g., PE, PP, PS). However, further catalyst development as well as adjustment of the reaction conditions holds promise to further reduce temperatures at which monomers may be obtained. Simultaneously, photocatalysts that can withstand high temperatures are essential to expand the range of polymers to ones with higher *T*_c_ values.

A wider range of polymers could be degraded to small molecules using photocatalysts to selectively cleave C‒C backbones (some examples of C‒X). Specific functional groups, such as hydroxy-substituted backbones for PCET, enabled selective photocatalytic C‒C backbone scission. Although most commodity polymers are not hydroxy-substituted, recent post-modification strategies introduced oxygen functionality, providing potential reactive sites for selective degradation processes^54,55^. Successful degradation of functional group-lacking polymer structures is more challenging but achievable with various HAT catalysts using UV to visible light irradiation. PS, with the lowest BDE of a C‒H bond in its backbone, has been extensively studied. To activate C‒C polymers with higher BDEs (C‒H) like PE, PP, PVC, and PVAc, only reactive/activated oxygen species have, so far, proven effective as HAT agents. After initial radical generation by HAT, oxygen was crucial for inducing radical fragmentation pathways, resulting in oxygenated small molecules. Predominantly, carboxylic acids were produced from various plastics (PS, PE, PP, PVAc). In the case of PS, milder reaction conditions, shorter reaction times, or choice of catalyst could also yield less oxidized products, such as benzaldehyde or acetophenone. Striking a balance between achieving high yields and avoiding overoxidation to CO_2_ remains a significant challenge for photooxidative degradation strategies.

Further catalyst development to tailor product selectivity as well as reduce reaction time is required to increase efficiency and consequently industrial potential. With regards to photooxidative degradation methods, an increased conversion efficiency is generally correlated with a decrease in selectivity and consequently a higher risk of overoxidation to undesirable CO_2_. Further development of HAT catalysts to improve site selectivity is therefore required, e.g., selective tertiary over secondary hydrogen abstraction, as was partially demonstrated for FeBr_3_ *vs* FeCl_3_ (see Fig. 6)^68^. However, achieving site-selective C‒H activation and oxidation in chemistry remains a challenging endeavor. In contrast, enzymatic reactions often exhibit remarkable selectivity. The naturally occurring enzyme Cytochrome P450, for example, can achieve site-selective oxidation to form alcohols^104^. Similar to Cytochrome P450, enhanced selectivity may be attained through supramolecular chemistry principles, where intermolecular interactions between substrate and catalyst allow for site selective reactivity^105^. Furthermore, enhancing the reactivity of hydrogen atom transfer (HAT) catalysts is desirable to circumvent the need for reactive oxygen species as HAT agents. This approach has the potential to mitigate overoxidation to CO_2_ by tailoring the oxygen content. Past studies (Table 3, entry 5) have used DFT calculations to simulate competing reaction pathways and transition states, particularly regarding peroxide formation and subsequent fragmentation mechanisms^69^. Such simulations can serve as a complementary tool to optimize reaction conditions. Screening various reaction conditions coupled with automated analysis techniques and machine learning may further enhance product selectivity and overall efficiency.

**Fig. 8:**
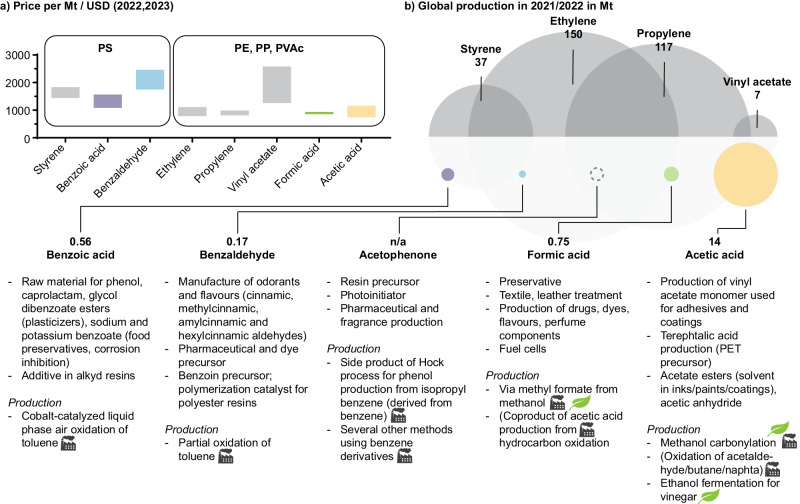
Comparison of major products from photocatalytic commodity plastic degradation (in color) to monomers (greyscale) of the respective polymer. **a** Chemical prices per ton in USD. The price ranges given are the highest and lowest prices of averaged regional prices from North America, APAC and Europe from 2022 to 2023^106^. **b** Global production in million tons (Mt) indicated by circle size^119^. The applications and commercial manufacturing methods of small molecule products are highlighted while noting their dependence on fossil fuels (factory-symbol) or sustainable production methods (leaf-symbol)^120–124^.

Specifically, when products other than monomers are produced, optimization for product value and market size is desirable. For example, the price/ton of benzophenone is estimated to be up to 2.4 times higher than e.g., benzoic acid, formic acid, or acetic acid, making it a more desirable target from an economic perspective (Fig. 8a)^106^. In terms of market size of the degradation products, acetic acid had the largest yearly production of 14 Mt due to its use for the manufacture of monomers vinyl acetate (for PVAc) and terephthalic acid (for PET) (Fig. 8b). These considerations may aid future catalyst development and the optimization of degradation processes, including reactor design and adjusting experimental parameters.

Producing monomers as degradation products is preferable for a circular economy (closed loop) and economically advantageous, as monomer prices generally exceeded those of other small molecule products (Fig. 8a). However, plastic waste degradation into small molecules, particularly organic acids through photooxidative processes, also yields valuable commodities applicable across various industries (Fig. 8b). In general, using plastics as feedstocks for chemical production offers a sustainable alternative to conventional fossil fuel-derived processes. For these sustainable approaches to be economically feasible their cost must be able to compete with established production methods which poses a clear challenge. Alternatively, obtaining more downstream products like surfactants and oils, or upcycled materials may provide a greater cost benefit, though this approach only postpones the waste recycling issue^60,91,107^. To assess the sustainability of the individual recycling technologies, a thorough life cycle assessment (LCA) is necessary to quantify overall greenhouse gas emissions. Hereby, resin production, plastic production, waste transport, and end of life processes, i.e., recycling methods, require consideration^108^. By reintroducing valuable chemicals into a circular system their production may be avoided which can be accounted for as avoided emissions^11^. In this context, the repeated recovery and reuse of employed catalysts also adds to the sustainability of photocatalytic approaches.

A major challenge of plastics recycling lies in the disparity between real-world plastic waste streams and the typically studied pristine materials for methodological development. Additives like plasticizers, flame retardants, UV stabilizers and colorants, among others, are generally added in the compounding process during plastics production^109^. These additives allow tailoring of the material properties and are generally proprietary knowledge resulting in materials of unknown compositions. In addition, real world plastic waste streams suffer from contamination and rely on efficient pre-sorting into different types of plastics. The result is a highly complex and unknown composition of plastic waste streams. An emerging industrial method to tackle this issue is pyrolysis, using high temperatures in an inert atmosphere to degrade waste into a mixture of products, including monomers, gases, oils, and char^11^.

In terms of photocatalytic methods discussed in this review, some photocatalytic examples have shown remarkable tolerance towards limited solubility, additives, or polymer mixtures. However, this topic has generally lacked attention requiring further systematic investigation. Photocatalytic methods are often hindered by poor light penetration either due to limited solubility or competing absorption from other components (i.e., UV stabilizers, colorants) with the photocatalyst. The presence of radical or excited state quenchers can also impede a photocatalyst’s efficiency. Therefore, the presence of additives may affect the efficacy of photocatalytic methods depending on their reactivity, optical properties, and concentration. While the effect of UV-absorbers may be circumvented with visible light responsive photocatalysts, the presence of colorants can negatively affect the visible light photocatalysts. Generally, a significant spectral overlap of the material and the photocatalyst’s absorption can reduce the method’s efficiency. Therefore, given the diverse range of additives, a comprehensive set of methods spanning various spectral ranges is essential to ensure effective photocatalytic degradation.

While these are just general considerations, different additives e.g., colorants (Table 3, entry 4) or food contaminants (Table 3, entry 8) have shown to affect photooxidative methods to varying degrees^61,64^. The selective degradation by PCET, however, tolerated a broader range of additives, such as colorants, carbon fiber or triphenyl phosphate^42^. Remarkably, this method enabled selective degradation of phenoxy resins, even in the presence of other commodity polymers (PS, PVC, polycarbonate, and PMMA) eliminating the need for prior sorting or purification towards these structures^41,42^. When selective transformations become challenging, for example for the degradation of polyolefins, highly reactive, but perhaps less selective methods may ensure efficient conversion of polymer-additive mixtures. For instance, the use of reactive oxygen species, as discussed for the oxidative degradation of several C‒C backbone plastics, is also generally applied to degrade a broad range of organic structures (including colorants)^110^. It therefore may offer the ability to ensure conversion at the cost of selectivity, similar to pyrolysis. The specific effects of the different types of additives and mixed plastic waste on recycling and degradation methods has not received much attention and requires significant advancement to cater for the complexity of real-world plastic waste streams.

Photocatalytic degradation methods of polyolefins remain scarce due to their challenging solubility and lack of functional groups. Alternatively, the application of heat and catalysts to degrade polyolefins under N_2_ or H_2_ atmosphere, i.e., by hydrogenolysis, cracking or metathesis, is more established^111^. These methods generally produce mixtures of alkanes of varying chain lengths which include waxes, oils, liquid fuels, lubricants, or gases. These products benefit from the already existing petrochemical infrastructure which allows the isolation of desired components or further conversion to desired products. Catalytic examples using oxidizing conditions and heat showed higher tolerance towards polymer mixtures allowing efficient conversion of PE-PP mixtures to high value fatty acids or PE-PS-PET waste mixtures to organic acids^91,112^. In the latter example, the mixture of organic acids was bio-converted using *Pseudomonas putida* yielding either *β*-ketoadipate or polyhydroxyalkanoates as products. The potential of this approach is further demonstrated by a recent example which used a bio-organism to convert benzoic acid from polystyrene to a fungal natural product and a biocontrol agent^113^.

Similarly, the production of organic acids from photooxidative degradation may benefit from being combined with additional biological conversions. Enzymes may also hold promise for both expanding the range of degradation products or simplifying oxidized product mixtures and are already successfully applied for the degradation of PET^114^. Direct enzymatic conversion of polyolefins is challenging due to the lack of functional groups in the backbone. A two-step process, combining initial photooxidative degradation of polyolefins with subsequent enzymatic conversion, could offer greater control over the degradation products. Mild degradation methods or combinations thereof provide opportunities to convert plastic waste unsuitable for thermal recycling, for example, when degradation occurs at temperatures lower than depolymerization.

Future developments in light driven plastics recycling ideally also focus on the use of non-toxic chemicals, have a high atom efficiency, minimal environmental impact, and produce valuable products of competitive price^115^. For industrial relevance, a drastic increase from the currently possible gram scale in photoflow reactors is required and will also rely on further development of photochemical reactors^116^. We are optimistic that as the momentum shifts further from selectively making to breaking bonds, the breadth of the photochemical toolbox will continue to produce highly selective degradation strategies.
